# Racial and Ethnic Disparities in Use of Novel Hormonal Therapy Agents in Patients With Prostate Cancer

**DOI:** 10.1001/jamanetworkopen.2023.45906

**Published:** 2023-12-01

**Authors:** Ting Martin Ma, Neeraj Agarwal, Brandon Mahal, Regina Barragan-Carrillo, Daniel Spratt, Matthew B. Rettig, Luca F. Valle, Michael L. Steinberg, Isla Garraway, Neha Vapiwala, Michael Xiang, Amar U. Kishan

**Affiliations:** 1 Department of Radiation Oncology, University of Washington, Seattle; 2 Division of Medical Oncology, Department of Internal Medicine, Huntsman Cancer Institute, University of Utah, Salt Lake City; 3 Department of Radiation Oncology, Sylvester Comprehensive Cancer Center, University of Miami Miller School of Medicine, Miami, Florida; 4 Department of Medical Oncology and Therapeutics Research, City of Hope Comprehensive Cancer Center, Duarte, California; 5 Department of Radiation Oncology, University Hospitals Seidman Cancer Center, Case Western Reserve University, Cleveland, Ohio; 6 Division of Hematology and Oncology, David Geffen School of Medicine at UCLA, Los Angeles, California; 7 Division of Hematology and Oncology, VA Greater Los Angeles Healthcare System, Los Angeles, California; 8 Department of Radiation Oncology, University of California, Los Angeles (UCLA), Los Angeles; 9 Department of Urology, UCLA, Los Angeles, California; 10 Jonsson Comprehensive Cancer Center, David Geffen School of Medicine at UCLA, Los Angeles, California; 11 Division of Urology, VA Greater Los Angeles Healthcare System, Los Angeles, California; 12 Department of Radiation Oncology, University of Pennsylvania, Philadelphia

## Abstract

**Importance:**

Novel hormonal therapy (NHT) agents have been shown to prolong overall survival in numerous randomized clinical trials for patients with advanced prostate cancer (PCa). There is a paucity of data regarding the pattern of use of these agents in patients from different racial and ethnic groups.

**Objective:**

To assess racial and ethnic disparities in the use of NHT in patients with advanced PCa.

**Design, Setting, and Participants:**

This cohort study comprised all men diagnosed with de novo advanced PCa (distant metastatic [M1], regional [N1M0], and high-risk localized [N0M0] per Systemic Therapy in Advancing or Metastatic Prostate Cancer: Evaluation of Drug Efficacy [STAMPEDE] trial criteria) with Medicare Part A, B, and D coverage between January 1, 2011, and December 31, 2017, in a Surveillance, Epidemiology, and End Results (SEER)–Medicare linked database including prescription drug records. Data analysis took place from January through May 2023.

**Exposures:**

Race and ethnicity (Black [non-Hispanic], Hispanic, White, or other [Alaska Native, American Indian, Asian, Pacific Islander, or not otherwise specified and unknown]) abstracted from the SEER data fields.

**Main Outcomes and Measures:**

The primary outcome was receipt of an NHT agent (abiraterone, enzalutamide, apalutamide, or darolutamide) using a time-to-event approach.

**Results:**

The study included 3748 men (median age, 75 years [IQR, 70-81 years]). A total of 312 (8%) were Black; 263 (7%), Hispanic; 2923 (78%), White; and 250 (7%) other race and ethnicity. The majority of patients had M1 disease (2135 [57%]) followed by high-risk N0M0 (1095 [29%]) and N1M0 (518 [14%]) disease. Overall, 1358 patients (36%) received at least 1 administration of NHT. White patients had the highest 2-year NHT utilization rate (27%; 95% CI, 25%-28%) followed by Hispanic patients (25%; 95% CI, 20%-31%) and patients with other race or ethnicity (23%; 95% CI, 18%-29%), with Black patients having the lowest rate (20%; 95% CI, 16%-25%). Black patients had significantly lower use of NHT compared with White patients, which persisted at 5 years (37% [95% CI, 31%-43%] vs 44% [95% CI, 42%-46%]; *P* = .02) and beyond. However, there was no significant difference between White patients and Hispanic patients or patients with other race or ethnicity in NHT utilization (eg, 5 years: Hispanic patients, 38% [95% CI, 32%-46%]; patients with other race and ethnicity: 41% [95% CI, 35%-49%]). Trends of lower utilization among Black patients persisted in the patients with M1 disease (eg, vs White patients at 5 years: 51% [95% CI, 44%-59%] vs 55% [95% CI, 53%-58%]). After adjusting for patient, disease, and sociodemographic factors in multivariable analysis, Black patients continued to have a significantly lower likelihood of NHT initiation (adjusted subdistribution hazard ratio, 0.76; 95% CI, 0.61-0.94, *P* = .01).

**Conclusions and Relevance:**

In this cohort study of Medicare beneficiaries with advanced PCa, receipt of NHT agents was not uniform by race, with decreased use observed in Black patients compared with the other racial and ethnic groups, likely due to multifactorial obstacles. Future studies are needed to identify strategies to address the disparities in the use of these survival-prolonging therapies in Black patients.

## Key Points

**Question:**

Are there racial and ethnic disparities in the use of novel hormonal therapy (NHT) agents in men with advanced prostate cancer?

**Findings:**

In this cohort study using a population-based cancer registry linked to prescription drug records for 3748 men diagnosed with advanced prostate cancer from 2011 to 2017, the rate of NHT initiation in Black patients was substantially lower than that in White patients, even after accounting for stage, disease characteristics, and socioeconomic factors.

**Meaning:**

The findings suggest that substantial, likely multifactorial barriers exist regarding racially equitable administration of NHT.

## Teaser

This cohort study uses data Surveillance, Epidemiology, and End Results–Medicare linked data to assess whether there are racial and ethnic disparities in use of novel hormonal therapy agents in patients with advance prostate cancer.

## Introduction

It is estimated that men in the US have a 12% lifetime risk of being diagnosed with prostate cancer (PCa).^1^ Modulation of the androgen signaling axis is a cornerstone in the treatment of advanced PCa.^2^ Androgen deprivation therapy (ADT) uses a gonadotropin-releasing hormone agonist or antagonist to suppress circulating androgen levels. Second-generation androgen receptor (AR) antagonists, such as enzalutamide, apalutamide, and darolutamide, have improved specificity and affinity for ARs compared with first-generation antagonists, and abiraterone acetate blocks extragonadal androgen biosynthesis.^3^ These agents, termed novel hormonal therapy (NHT), when combined with ADT, more effectively inhibit androgen-dependent signaling than ADT alone or ADT combined with first-generation AR antagonists.^3^ Multiple large randomized clinical trials have demonstrated increased overall survival, leading to a series of approvals by the US Food and Drug Administration (FDA) for metastatic and high-risk nonmetastatic PCa.^4,5,6,7,8^ However, despite the proven benefits of NHT agents, their use in the general population remains poorly understood, particularly in the context of equitable access to these medications across different race and ethnicity groups.^9^ Herein, we investigated disparities in prescription patterns of NHT according to patients’ race and ethnicity in the US using a Surveillance, Epidemiology, and End Results (SEER)–Medicare linked database including prescription drug records.

## Methods

This cohort study using 2020 SEER-Medicare linked data (considered a limited data set per Health Insurance Portability and Accountability Act regulations) was deemed exempt from approval by the UCLA institutional review board, with a waiver of informed consent per 45 CFR §46. Our study followed the Strengthening the Reporting of Observational Studies in Epidemiology (STROBE) reporting guideline.

We identified patients diagnosed from January 1, 2011 (first year that abiraterone was FDA approved in the US), through December 31, 2017 (last year of diagnosis available for the 2020 database linkage). Patients with prostatic adenocarcinoma with no previous cancer diagnosis and with distant metastatic (M1), regional (N1M0), or high-risk localized (N0M0) PCa per Systemic Therapy in Advancing or Metastatic Prostate Cancer: Evaluation of Drug Efficacy (STAMPEDE) trial criteria^10^ were included to broadly encompass patients most likely to be considered for and offered NHT. Patients were also required to have known length of follow-up, Medicare Parts A and B coverage, and enrollment in Part D at the time of diagnosis. Patients had to initiate ADT (gonadotropin-releasing hormone agonist or antagonist) within 180 days of diagnosis. The primary outcome was administration of an NHT agent (abiraterone, enzalutamide, apalutamide, or darolutamide) measured through December 31, 2019 (latest follow-up for the 2020 database). Data analysis was from January through May 2023. Dates of publications of key supporting clinical trials and FDA approval for NHT agents in this study for various indications are shown in the eTable in Supplement 1.

Race and ethnicity were determined based on the SEER data fields and classified into mutually exclusive categories of Black (non-Hispanic), Hispanic, White, or other (Alaska Native, American Indian, Asian, Pacific Islander, or not otherwise specified and unknown). Other variables included patient factors and disease characteristics at diagnosis (eMethods in Supplement 1).

### Statistical Analysis

Baseline characteristics were compared using the Kruskal-Wallis test and the χ^2^ test. Use of NHT was analyzed using a time-to-event approach, measured as the time from initial diagnosis to the first administration of NHT and graphed as cumulative incidence, with death without NHT as a competing event. In the competing risks analysis, loss of part D coverage (if occurring prior to prescription of NHT) was treated using censoring (the same as for patients alive at last follow-up without being prescribed NHT). Thus, the time-to-event analysis accounted for possible loss of part D coverage. Curves were stratified by race and ethnicity and compared using the Gray test. Multivariable analysis was performed using Fine-Gray regression. Computations were performed using MATLAB, version R2022a (MathWorks) and R, version 4.2.0 (R Project for Statistical Computing) with the cmprsk package, version 2.2-10. Two-sided *P* < .05 was considered statistically significant.

## Results

The study included 3748 men (median age, 75 years [IQR, 70-81 years]) (eFigure 1 in Supplement 1). A total of 312 (8%) were Black; 263 (7%), Hispanic; 2923 (78%), White; and 250 (7%), other race and ethnicity. There were significant differences among racial and ethnic groups in baseline characteristics, such as census tract poverty level, socioeconomic quintile, T category, and initial prostrate-specific antigen level at diagnosis (Table 1).

**Table 1.** Baseline Characteristics of Patients Included in the Study

Characteristic	Patients^a^				*P* value
	Black (n = 312)	Hispanic (n = 263)	White (n = 2923)	Other (n = 250)^b^	
Age, median (IQR)	73 (69-78)	75 (70-80)	75 (71-81)	76 (71-82)	<.001
Marital status					
Married	125 (40)	166 (63)	1941 (66)	177 (71)	<.001
Other	187 (60)	97 (37)	982 (34)	73 (29)	
Year of diagnosis, median (IQR)	2014 (2013-2016)	2015 (2013-2016)	2015 (2013-2016)	2015 (2013-2016)	.13
Urban or rural					
Metropolitan	276 (88)	241 (92)	2324 (80)	233 (93)	<.001
Nonmetropolitan	36 (12)	22 (8)	599 (20)	17 (7)	
Poverty level, %					
<5	27 (9)	45 (17)	748 (26)	50 (20)	<.001
5-10	38 (12)	49 (19)	840 (29)	81 (32)	
11-20	62 (20)	84 (32)	817 (28)	73 (29)	
>20	185 (59)	85 (32)	518 (18)	46 (18)	
SES quintile^c^					
Fifth	26 (8)	47 (18)	961 (33)	99 (40)	<.001
Fourth	47 (15)	48 (18)	735 (25)	54 (22)	
Third	41 (13)	53 (20)	510 (17)	42 (17)	
Second	50 (16)	55 (21)	420 (14)	27 (11)	
First	148 (47)	60 (23)	297 (10)	28 (11)	
T category					
T1	109 (35)	69 (26)	676 (23)	67 (27)	<.001
T2	62 (20)	58 (22)	681 (23)	42 (17)	
T3-4	141 (45)	136 (52)	1566 (54)	141 (56)	
Gleason score					
6-7	36 (12)	32 (12)	256 (9)	15 (6)	.06
8	81 (26)	77 (29)	739 (25)	70 (28)	
9-10	195 (63)	154 (59)	1928 (66)	165 (66)	
PSA level, median (IQR), ng/mL^d^	98 (39-98)	56 (19-98)	44.9 (13-98)	55 (13-98)	<.001
Disease extent^e^					
M1	189 (61)	145 (55)	1663 (57)	138 (55)	.01
N1M0	22 (7)	39 (15)	427 (15)	30 (12)	
N0M0	101 (32)	79 (30)	833 (28)	82 (33)	
Charlson Comorbidity Index score					
0	127 (41)	114 (43)	1396 (48)	93 (37)	<.001
1-2	115 (37)	86 (33)	1029 (35)	108 (43)	
≥3	70 (22)	63 (24)	498 (17)	49 (20)	

Abbreviations: PSA, prostate-specific antigen; SES, socioeconomic status.

SI conversion: To convert PSA level to micrograms per liter, multiply by 1.0.

^a^ Data are presented as number (percentage) of patients unless otherwise indicated.

^b^ Other includes Alaska Native, American Indian, Asian, Pacific Islander, or not otherwise specified and unknown.

^c^ Based on the Yost Index, in which fifth is the highest quintile and first is the lowest.

^d^ Prostate-specific antigen values greater than 98 ng/mL are recorded as 98 ng/mL in the Surveillance, Epidemiology, and End Results database.

^e^ Distant metastatic (M1), regional (N1M0), and high-risk localized (N0M0) per Systemic Therapy in Advancing or Metastatic Prostate Cancer: Evaluation of Drug Efficacy (STAMPEDE) trial criteria.

Overall, the majority of patients had M1 disease (2135 [57%]) followed by high-risk N0M0 (1095 [29%]) and N1M0 (518 [14%]) disease. A total of 1358 patients (36%) received at least 1 administration of NHT: abiraterone was the first agent for 825 patients (61%) and enzalutamide for 514 (38%). Only 19 patients (1%) received darolutamide or apalutamide as their first NHT administration. The median follow-up was 3.5 years (IQR, 2.3-5.4 years) in the entire cohort.

Numerically, White patients had the highest 2-year NHT utilization rate (27%; 95% CI, 25%-28%) followed by Hispanic patients (25%; 95% CI, 20%-31%) and patients with other race and ethnicity (23%; 95% CI, 18%-29%), with Black patients having the lowest rate (20%; 95% CI, 16%-25%). Black patients also had significantly lower utilization compared with White patients, which persisted at 5 years (37% [95% CI, 31%-43%] vs 44% [95% CI, 42%-46%]; *P* = .02) and beyond (Figure). However, there was no significant difference between White patients and Hispanic patients or patients with other race and ethnicity (eg, 5 years: Hispanic patients, 38% [95% CI, 32%-46%]; patients with other race and ethnicity: 41% [95% CI, 35%-49%]).

**Figure.**
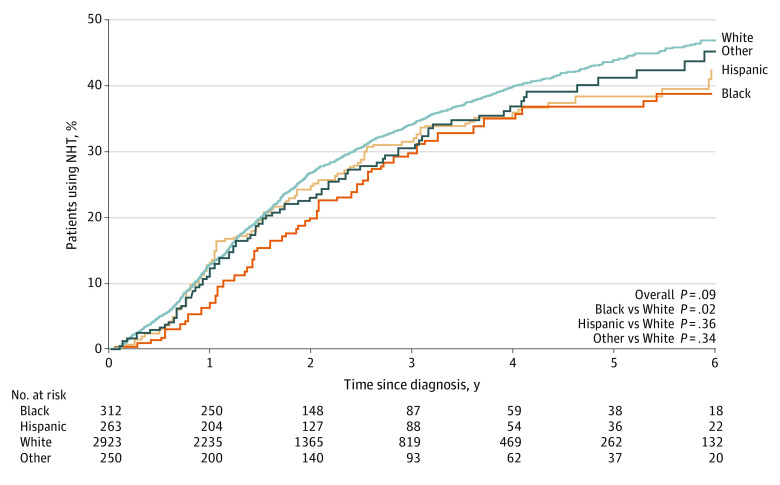
Cumulative Incidence of Novel Hormonal Therapy (NHT) Use in Patients With Advanced-Stage Prostate Cancer Stratified by Race and Ethnicity Two-sided *P* < .05 was considered statistically significant.

In subgroup analysis, trends of lower utilization among Black patients persisted in patients with M1 (eg, vs White patients at 5 years: 51% [95% CI, 44%-59%] vs 55% [95% CI, 53%-58%]) (eFigure 2 in Supplement 1) and high-risk N0M0 (eg, vs White patients at 5 years: 12% [95% CI, 6%-22%] vs 23% [95% CI, 19%-27%]) (eFigure 3 in Supplement 1) disease. It was not possible to perform a subgroup analysis for patients with N1M0 disease given the small sample size.

After adjusting for patient factors, disease, baseline comorbidity status, and sociodemographic factors in the multivariable model, Black patients continued to be significantly less likely to receive NHT (adjusted subdistribution hazard ratio [SHR], 0.76; 95% CI, 0.61-0.94; *P* = .01). There was no significant difference for Hispanic patients compared with White patients (adjusted SHR, 0.99; 95% CI, 0.79-1.24; *P* = .95) or for patients with other race and ethnicity compared with White patients (adjusted SHR, 0.91; 95% CI, 0.73-1.14; *P* = .42) (Table 2). Factors associated with increased likelihood of receiving NHT included White race (compared with Black race), younger age at diagnosis, Gleason score of 9 or 10, higher prostate-specific antigen level, M1 disease at diagnosis, lower Charlson Comorbidity Index score, married status, and higher socioeconomic status.

**Table 2.** Multivariable Analysis of Variables Associated With Receipt of Novel Hormonal Therapy Agents

Variable	Adjusted SHR (95% CI)	*P* value
Race/ethnicity		
Black	0.76 (0.61-0.94)	.01
Hispanic	0.99 (0.79-1.24)	.95
White	1 [Reference]	NA
Other^a^	0.91 (0.73-1.14)	.42
Age, per 1 y	0.97 (0.97-0.98)	<.001
Marital status		
Married	1 [Reference]	NA
Other	0.86 (0.76-0.97)	.01
Year of diagnosis (per 1 y)	1.15 (1.11-1.18)	<.001
Urban or rural		
Metropolitan	1 [Reference]	NA
Nonmetropolitan	0.84 (0.71-0.99)	.04
Poverty level, %		
<5	1 [Reference]	NA
5-10	1.09 (0.92-1.29)	.32
11-20	1.24 (1.02-1.50)	.03
>20	1.11 (0.87-1.42)	.40
SES quintile^b^		
Fifth	1 [Reference]	NA
Fourth	0.97 (0.82-1.14)	.68
Third	0.68 (0.55-0.84)	<.001
Second	0.75 (0.60-0.95)	.02
First	0.80 (0.61-1.05)	.11
T category		
T1	1 [Reference]	NA
T2	0.91 (0.78-1.07)	.24
T3-4	1.00 (0.87-1.14)	.98
Gleason score		
6-7	1 [Reference]	NA
8	1.19 (0.94-1.50)	.14
9-10	1.80 (1.47-2.21)	<.001
PSA level, per 1 ng/mL	1.01 (1.00-1.01)	<.001
Disease extent^c^		
M1	1 [Reference]	NA
N1M0	0.51 (0.43-0.61)	<.001
N0M0	0.29 (0.24-0.34)	<.001
Charlson Comorbidity Index score		
0	1 [Reference]	NA
1-2	0.86 (0.76-0.97)	.01
≥3 or higher	0.74 (0.63-0.87)	<.001

Abbreviations: NA, not applicable; PSA, prostate-specific antigen; SES, socioeconomic status; SHR, subdistribution hazard ratio.

^a^ Other includes Alaska Native, American Indian, Asian, Pacific Islander, or not otherwise specified and unknown.

^b^ Based on the Yost Index, in which fifth is the highest quintile and first is the lowest.

^c^ Distant metastatic (M1), regional (N1M0), and high-risk localized (N0M0) per Systemic Therapy in Advancing or Metastatic Prostate Cancer: Evaluation of Drug Efficacy (STAMPEDE) trial criteria.

## Discussion

Using a national cancer registry linked to prescription drug records, we found that Black men with advanced PCa were less likely to receive NHT than White men despite proven clinical benefit of these agents. In the US, Black men are 1.5 times more likely to be diagnosed with PCa and 2.4 times more likely to die of PCa than White men.^11^ Black men also disproportionately present at more advanced stages of disease,^12^ with exacerbation of this scenario by the US Preventive Services Task Force recommendation against prostrate-specific antigen screening in 2012.^13^ This evidence makes use of NHT in this patient population particularly important given its substantial clinical benefit. However, we found a concerning lower rate of utilization of NHT in Black Medicare beneficiaries in the general population. Intriguingly, this disparity was specific to Black patients and was not observed with other racial and ethnic groups. The underlying causes for this disparity, which persisted after adjusting for disease characteristics and sociodemographic variables, are unknown and are likely multifactorial, possibly including barriers to care, financial toxicity, lack of health care practitioner education in geographic regions serving predominantly Black beneficiaries, and implicit bias of health care practitioners.^14,15^ We observed racial and ethnic disparities despite all patients in the study by virtue of eligibility criteria being Medicare beneficiaries, which has improved access to health care for older patients. Note that Medicare Part D is not administered directly by the federal government but is privatized via insurance companies that contract with the government, which could also have been one of the factors that contributed to the disparity observed.

### Limitations

The current study has several limitations. First, our study included only patients diagnosed with de novo advanced PCa since only the initial stage at diagnosis is available in SEER. Second, castration-resistant status and details regarding first-line chemotherapy receipt were not available. Third, clinical indications and FDA approvals for NHT evolved over the study period. Nonetheless, we observed similar findings in the subgroup analysis of patients with M1 disease, for which the FDA provided the earliest approval for NHT. Furthermore, evolving clinical indications and FDA approvals would not affect Black men differently from White men and cannot explain the disparity in NHT use observed in this study. Finally, the current analysis is based on practices during early adoption of NHT, and future studies are needed to draw conclusions regarding current practice in 2023.

## Conclusions

In this cohort study using a population-based cancer registry linked to prescription drug records, there was a substantially lower rate of utilization of NHT in Black men with advanced PCa compared with White men with PCa. Future studies are needed to uncover underlying causes and to systematically address these issues for more equitable care.

## Notes

Supplement 1.
eMethods.
**eTable.** Major clinical trials, publication date, FDA approval date and NCCN guideline recommendation status of novel hormonal therapy agents **eFigure 1.** Cohort Identification **eFigure 2.** NHT utilization in White compared to Black patients in the M1 subgroup **eFigure 3.** NHT utilization in White compared to Black patients in the high-risk N0M0 subgroup

Supplement 2.
Data Sharing Statement
