# Supplementary material for: Racial and Ethnic Disparities in Use of Novel Hormonal Therapy Agents in Patients With Prostate Cancer
Source: JAMA Netw Open. 2023 Dec 1;6(12):e2345906. doi: 10.1001/jamanetworkopen.2023.45906 (PMC10692845; doi:10.1001/jamanetworkopen.2023.45906)
Supplement: Supplement 1. — eMethods. eTable. Major clinical trials, publication date, FDA approval date and NCCN guideline recommendation status of novel hormonal therapy agents eFigure 1. Cohort Identification eFigure 2. NHT utilization in White compared to Black patients in the M1 subgroup eFigure 3. NHT utilization in White compared to Black patients in the high-risk N0M0 subgroup [file jamanetwopen-e2345906-s001.pdf]

Supplemental Online Content

Ma TM, Agarwal N, Mahal B, et al. Racial and ethnic disparities in use of novel hormonal therapy agents in patients with prostate cancer. *JAMA Netw Open.* 2023;6(12):e2345906. doi:10.1001/jamanetworkopen.2023.45906

eMethods.

eTable. Major clinical trials, publication date, FDA approval date and NCCN guideline recommendation status of novel hormonal therapy agents

eFigure 1. Cohort Identification

eFigure 2. NHT utilization in White compared to Black patients in the M1 subgroup

eFigure 3. NHT utilization in White compared to Black patients in the high-risk N0M0 subgroup

This supplemental material has been provided by the authors to give readers additional information about their work.

© 2023 Ma TM et al. *JAMA Network Open.*

eMethods

Data Source

The Surveillance, Epidemiology, and End Results (SEER) registry captures all incident cancers from 17 regional registries corresponding to 30% of the United States population. The SEER-Medicare database links patients in SEER with their Medicare claims, which allows the determination of specific diagnoses and procedures across time using International Classification of Diseases (ICD) and Healthcare Common Procedure Coding System (HCPCS) codes. Medicare provides health insurance for the majority of Americans over age 65, and patients enrolled in Medicare Part D have oral drug prescription data available. The 2020 SEER-Medicare database linkage was used in this study. All data were de-identified. The study was deemed exempt by the institutional review board.

Cohort Identification Procedure

Patients were included if they were diagnosed from year 2011 (the first year that abiraterone was FDA-approved in the United States) through the end of year 2017 (the last year of diagnosis available for the 2020 database linkage). All patients were required to have histologically confirmed prostatic adenocarcinoma with no previous cancer diagnosis. To ensure adequate capture of claims data, patients were further required to have a known length of follow-up, Medicare Part A and B coverage and no health maintenance organization (HMO) within 12 months of diagnosis, enrollment in Medicare for age only, and enrollment in Medicare Part D at the time of diagnosis.

Patients were further filtered to identify a population with disease features most likely to be clinically eligible for and offered novel hormonal therapy (NHT) by their provider. These inclusion criteria were patients with distant metastatic (M1), regional (N1M0), or high-risk localized (N0M0) prostate cancer (defined according to STAMPEDE criteria as at least 2 of the following 3 present: T3-4; Gleason 8-10; and/or PSA ≥ 40 ng/ml) at the time of diagnosis. These criteria were chosen since they are the most recognized indications for the utilization of NHT and are supported by high-level clinical trial evidence. Finally, to be included in this study, patients had to initiate ADT (GnRH agonist or antagonist) within 180 days of diagnosis. ADT was ascertained using the HCPCS codes J9217-J9219 and J1950 (leuprolide), J9202 (goserelin), J9155 (degarelix), J3315 (triptorelin), and J9225 (histrelin).

Besides race/ethnicity, variables in the analysis included patient factors at diagnosis (age, marital status, year of diagnosis, urban/rural residence, census tract poverty level, and Yost Index of socioeconomic status) and disease characteristics at diagnosis (T category, Gleason score, PSA and disease extent).

© 2023 Ma TM et al. *JAMA Network Open.*

Supplemental Tables

eTable . Major clinical trials, publication date, FDA approval date and NCCN guideline recommendation status of novel hormonal therapy agents

NHT agent	Context	Trial name	Trial publication date*	FDA approval date	NCCN guideline recommendation
Abiraterone	**mCRPC (after prior docetaxel)**	COU-AA-301	5/26/2011	4/28/2011	yes
	**mCRPC (prior to chemotherapy)**	COU-AA-302	1/10/2013	12/10/2012	yes
	**High-risk locally advanced****	STAMPEDE	7/27/2017	n/a	no
	**Node positive, non-metastatic (N1M0)**	STAMPEDE	7/27/2017	n/a	yes
	**mCSPC**	LATITUDE	4/12/2019	2/7/2018	yes
		STAMPEDE	7/27/2017		
Enzalutamide	**mCRPC (after prior docetaxel)**	AFFIRM	9/27/2012	8/31/2012	yes
	**mCRPC (prior to chemotherapy)**	PREVAIL	7/31/2014	n/a	yes
		TERRAIN	1/13/2016		
		STRIVE	1/15/2016		
	**nmCRPC**	PROSPER	6/28/2018	7/13/2018	yes
	**mCSPC**	ENZAMET	7/11/2019	12/16/2019	yes
		ARCHES	7/22/2019		
	mCRPC (HRR gene-mutated)	TALAPRO-2	7/4/2023	6/20/2023¶	yes
Apalutamide	**nmCRPC**	SPARTAN	4/12/2018	2/14/2018	yes
	**mCSPC**	TITAN	7/4/2019	9/17/2019	yes
Darolutamide	**nmCRPC**	ARAMIS	3/28/2019	7/30/2019	yes
	mCSPC	ARASENS	3/24/2022	8/5/2022	yes

Note: The context/indications in bold are ones within the study period of the current study (2011- end of 2019).

NHT, novel hormonal therapy; mCRPC, metastatic castration-resistant prostate cancer; mCSPC, metastatic castration-sensitive prostate cancer; nmCRPC, non-metastatic castration-resistant prostate cancer FDA, food and drug administration ; NCCN, National Comprehensive Cancer Network

* Trial publication date refers to the official publication date of the trial in the medical journal. The online publication date of the article is typically a few months earlier and the trial results may have been presented in academic conferences well before that, albeit not in the final format.
** at least two of the three high-risk prognostic factors (Gleason score of ≥8, presence of three or more lesions on bone scan, or presence of measurable visceral metastasis except lymph node metastasis)
¶ FDA approved talazoparib, in combination with enzalutamide, for the treatment of adult patients with homologous recombination repair (HRR) gene-mutated metastatic castration-resistant prostate cancer (mCRPC)

© 2023 Ma TM et al. *JAMA Network Open.*

Supplemental Figures

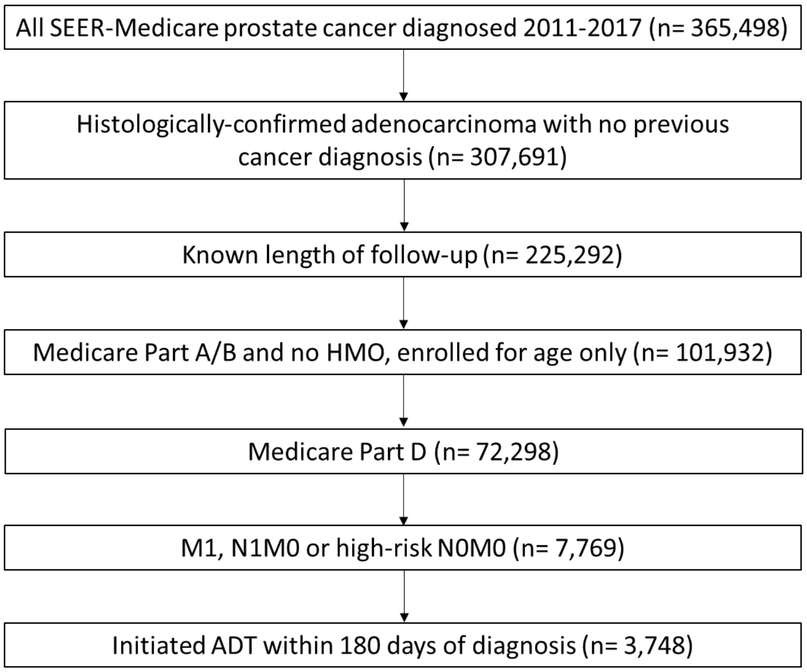

eFigure 1. Cohort Identification. SEER, Surveillance, Epidemiology, and End Results; HMO, health maintenance organization; ADT, androgen-deprivation therapy.

© 2023 Ma TM et al. *JAMA Network Open.*

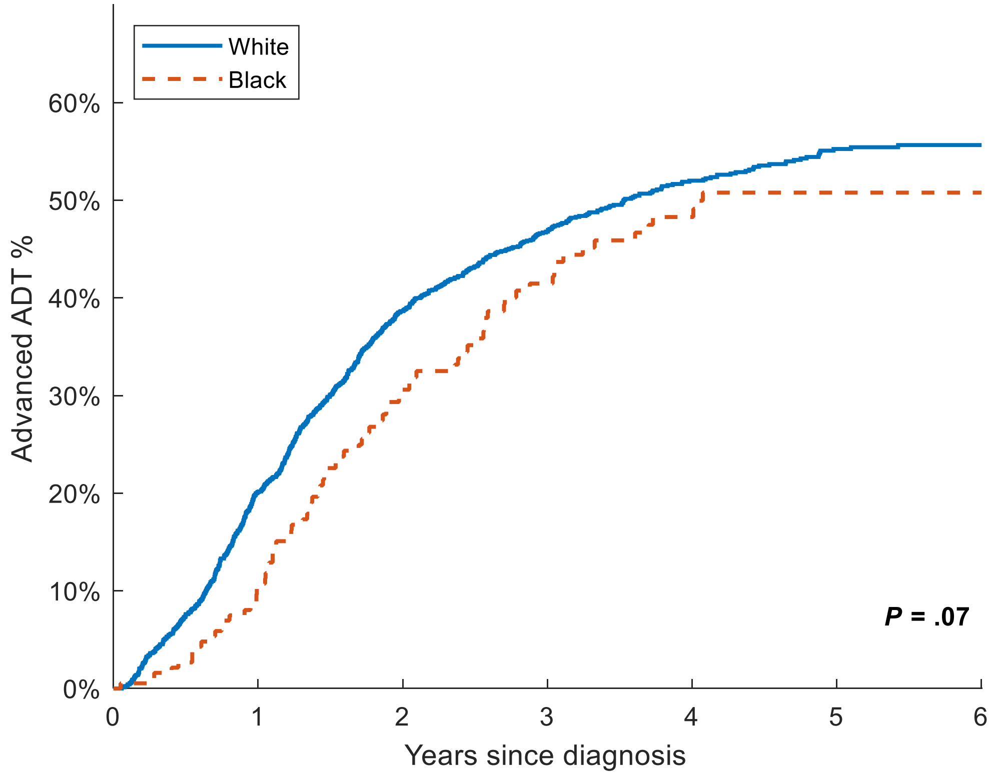

White:	1663	1083	544	280	129	53	23
Black:	189	137	65	35	21	*	*

eFigure 2. NHT utilization in White compared to Black patients in the M1 subgroup. Asterisk (*) denotes masking of small sample size to protect patient anonymity.

© 2023 Ma TM et al. *JAMA Network Open.*

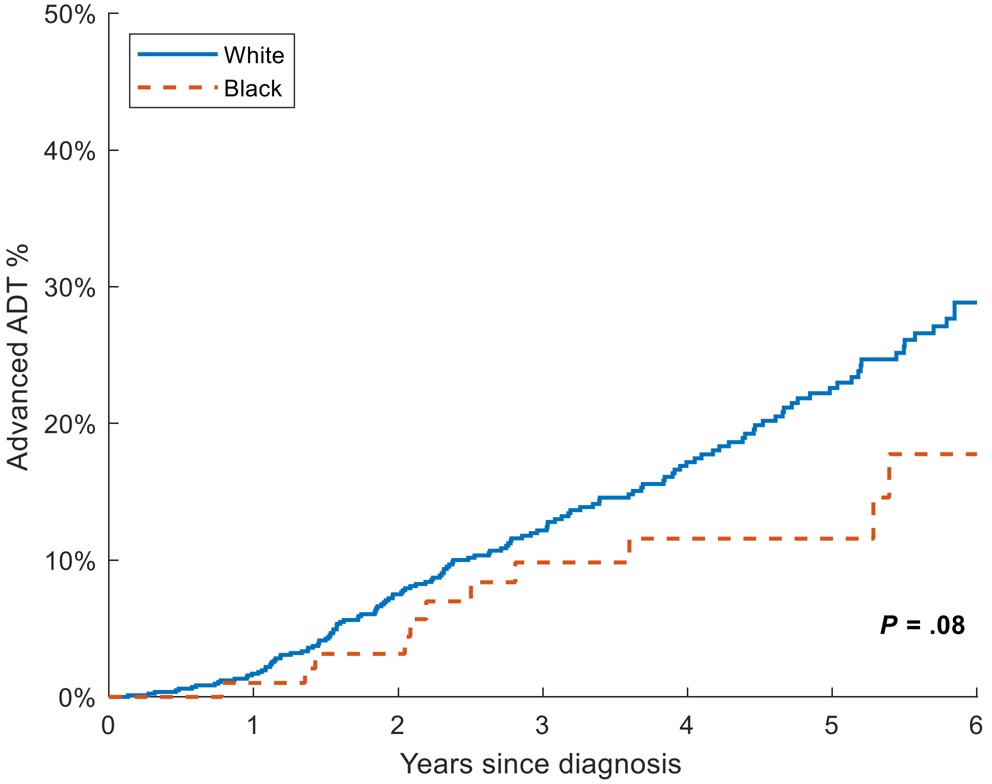

White:	833	765	550	368	235	142	74
Black:	101	*	68	47	35	22	11

eFigure 3. NHT utilization in White compared to Black patients in the high-risk N0M0 subgroup. Asterisk (*) denotes masking to protect patient anonymity due to small differences by subtraction to adjacent numbers-at-risk.

© 2023 Ma TM et al. *JAMA Network Open.*
