# Supplementary material for: Racial and Ethnic Disparities in Use of Novel Hormonal Therapy Agents in Patients With Prostate Cancer
Source: JAMA Netw Open. 2023 Dec 1;6(12):e2345906. doi: 10.1001/jamanetworkopen.2023.45906 (PMC10692845; doi:10.1001/jamanetworkopen.2023.45906)
Supplement: Supplement 2. — Data Sharing Statement [file jamanetwopen-e2345906-s002.pdf]

Data Sharing Statement

Ma. Racial and Ethnic Disparities in Use of Novel Hormonal Therapy Agents in Patients With Prostate Cancer. *JAMA Netw Open*. Published December 01, 2023. doi:10.1001/jamanetworkopen.2023.45906

Data
Data available: No
